# Ultrasound-Guided Needle Biopsy as an Alternative to Chamberlain’s Mediastinotomy and Video-Assisted Thoracoscopic Surgery (VATS) in the Diagnosis of Anterior Mediastinal Neoformations: A Retrospective Analysis

**DOI:** 10.3390/jcm12155070

**Published:** 2023-08-01

**Authors:** Federico Vischia, Giacomo Di Maio, Simona A. I. Ferrero, Elio Rolfo, Luca Scaglione, Riccardo Cristofori, Enrico Ruffini, Bartolomeo Lorenzati, Andrea Landi, Domenico Novero, Simona Capello, Giulia Schivazappa, Giorgio Limerutti, Arianna Ferro, Marilena Durazzo

**Affiliations:** 1 SCU Internal Medicine 3, Molinette Hospital, Città della Salute e della Scienza, 10126 Turin, Italy; 2 Department of Medical Sciences, University of Turin, 10124 Turin, Italy; 3 SC Internal Medicine 5, Molinette Hospital, Città della Salute e della Scienza, 10126 Turin, Italy; 4 SCU Thoracic Surgery, Molinette Hospital, Città della Salute e della Scienza, 10126 Turin, Italy; 5 SC Emergency Medicine, SS Annunziata Hospital, 12038 Savigliano, Italy; 6 Unit of Pathological Anatomy, Quality and Safety of Diagnosis and Treatment, Città della Salute e della Scienza, 10126 Turin, Italy; 7 SC Radiology 2, Molinette Hospital, Città della Salute e della Scienza, 10126 Turin, Italy

**Keywords:** mediastinal diseases, biopsy, diagnosis, ultrasonography

## Abstract

(1) Background: The prompt diagnosis of anterior mediastinal lesions is a challenge due to their often being categorized as malignant tumours. Ultrasound-guided Transthoracic Core Needle Biopsy (US–TCNB) is an innovative technique that is arousing increasing interest in clinical practice. However, studies in this area are still scarce. This study aims to compare the diagnostic accuracy and complication rate of US–TCNB with those of traditional surgical methods—Anterior Mediastinotomy and Video Assisted Thoracoscopic Surgery (VATS)—in patients with anterior mediastinal lesions. (2) Methods: This retrospective study involved patients evaluated between January 2011 and December 2021 who had undergone US–TCNB at the Interdepartmental Unit of Internal and Interventional Ultrasound, Molinette Hospital, Città della Salute e della Scienza, Turin, Italy. Personal data, diagnostic questions, and technical information concerning the bioptic procedure, periprocedural complications and histological reports were collected. (3) Results: Eighty-three patients were included in the analysis. Histological examination was performed in 78 cases, with an overall diagnostic accuracy of 94.0% (sensitivity 94%; specificity 100%). Only in 5 patients was a diagnosis not achieved. Complications occurred in 2 patients who were quickly identified and properly treated without need of hospitalization. The accuracy of US–TCNB was comparable to the performance of the main traditional diagnostic alternatives (95.3% for anterior mediastinotomy, and 98.4% for VATS), with a much lower complication rate (2.4% vs. 3–16%). The outpatient setting offered the additional advantage of saving resources. (4) Conclusions: a US-guided needle biopsy can be considered effective and safe, and in the near future it may become the procedure of choice for diagnosing anterior mediastinal lesions in selected patients.

## 1. Introduction

Mediastinal lesions are quite rare with a prevalence ranging from 0.7 to 0.9% [1]. More than 60% of these occur in the anterior compartment and include thymomas, about 35%; lymphomas, 25% (13% Hodgkin’s and 12% non-Hodgkin’s); thyroid tumours, 15%; germ cell tumours, 10%; and other benign masses, 10–15% [2,3,4]. Age and sex are the two main factors in the epidemiology of mediastinal pathologies [3]. Among older patients, thyroid and thymus neoplasms represent more than two-thirds of anterior mediastinal lesions, while in younger patients, epidemiology is influenced by sex, with a preponderance of lymphomas among females [3].

As is the case with other neoplastic diseases, prompt diagnosis is a challenge for patient prognosis. However, clinical signs are often non-specific and some patients are completely asymptomatic [5]. If present, symptoms can be localized or systemic. The former is related to the mass effect, which causes compression or infiltration of close structures (e.g., dyspnoea, chest pain, dysphagia). The latter is associated with the production of auto-antibodies, cytokines or endocrine molecules (e.g., weight loss, night sweats, fever) [6]. In addition, most laboratory blood tests are not very specific because serum tumour biomarkers are only available in a few cases. Thus, instrumental imaging examinations are almost always necessary for confirming the presence of mediastinal lesions [7]. Chest X-ray, Computed Tomography (CT), Magnetic Resonance Imaging (MRI) and Positron Emission Tomography (PET) are the most commonly used imaging techniques for mediastinal lesion detection and evaluation (size, location, metabolic activity) [8,9,10]. Nevertheless, imaging and clinical presentation are often not sufficient; therefore, a biopsy and histological examination are frequently required to confirm the diagnosis.

A biopsy can be performed surgically or percutaneously. Anterior Parasternal Mediastinotomy, also known as Chamberlain procedure, is the main surgical technique. It is widely used and it is considered the “gold standard” by many international guidelines (including ESMO—European Society for Medical Oncology and IAMO—Italian Association of Medical Oncology) [11,12,13,14]. However, it is an invasive and quite expensive procedure. Therefore, Video-Assisted Thoracoscopy (VATS) is often used as an alternative technique in clinical practice because of lower postoperative pain, shorter hospital stays and reduced overall costs [15,16]. Nevertheless, both procedures are carried out under general anaesthesia and may cause potentially life-threatening complications (e.g., bleeding, pneumothorax, surgical site infection, nerve injury and impaired respiratory function).

Hence, in recent years, percutaneous methods such as the Ultrasound-guided Transthoracic Core Needle Biopsy (US–TCNB) have gained increasing interest. This innovative technique allows tissue samples to be collected in real time guided by ultrasound. Unlike the other approaches described above, US–TCNB can be performed on an outpatient basis and requires only local anaesthesia (apart from anxiolytic drugs such as short-acting benzodiazepines, as needed). For this reason, US–TCNB needs fewer resources and can be performed on patients who are not eligible for surgery [17]. Moreover, the US-guided technique offers further diagnostic opportunities: the use of B-mode ultrasound integrated by Doppler modules allows easy study of the lesions’ anatomical features, vascularization and relationships with close vital structures; and probe handling enables modification of the scanning angle to show the mass from different points of view [18,19].

Furthermore, US–TCNB has many advantages over other widespread percutaneous techniques such as Trans-Bronchial Needle Aspirations (TBNA) and CT-guided core needle biopsy (TC–CNB). In fact, compared to TBNA, US–TCNB allows the extraction of a larger tissue sample enabling it to provide histological structure information, which is of the utmost importance for complex classification tumours (e.g., lymphomas or thymomas) [20]. Similarly, US-TCNB provides both economic and safety benefits compared to CT-guided core needle biopsy. In fact, although TC-CNB is the most widely known and used percutaneous method, it requires ionizing radiation, which not only puts patients at greater risk, but also increases costs [21], making US-TCNB a safer and cheaper technique. High cost also characterizes another percutaneous practice: MRI-guided biopsy. For this reason, despite good image quality and low invasiveness, this technique is rarely used in clinical practice or research [22].

Nevertheless, research into the use of US-guided core needle biopsy is scarce and those studies that exist often involve only a few patients [18,19,23,24].

Therefore, this study aims to evaluate the diagnostic accuracy and the complication rate of US–TCNB in patients with anterior mediastinal lesions. The results were compared to those obtained by Anterior Mediastinotomy and VATS to identify the main advantages and disadvantages including costs.

## 2. Materials and Methods

This retrospective study involved all patients who underwent US–TCNB at Interdepartmental Unit of Internal and Interventional Ultrasound, Molinette Hospital, Città della Salute e della Scienza of Turin, Italy. A search of the Molinette Hospital database was carried out for the period January 2011 to December 2021.

Subjects with suspected mediastinal lesions were selected. Among these, patients with non-deep site biopsies, non-US-guided procedures, mislabelled lesions and non-mediastinal lesions were excluded.

Demographic information, diagnostic questions, periprocedural complications and anatomo-pathological reports were drawn from medical records.

Before the procedure, each patient underwent a radiological study by CT contrast media and thoracic ultrasound to localize the target mass and characterize its anatomical relationships with the main structures and vessels of the mediastinum. Pleural contact with the lesion was considered a basic requirement for the US–TCNB procedure.

**Figure 1:**
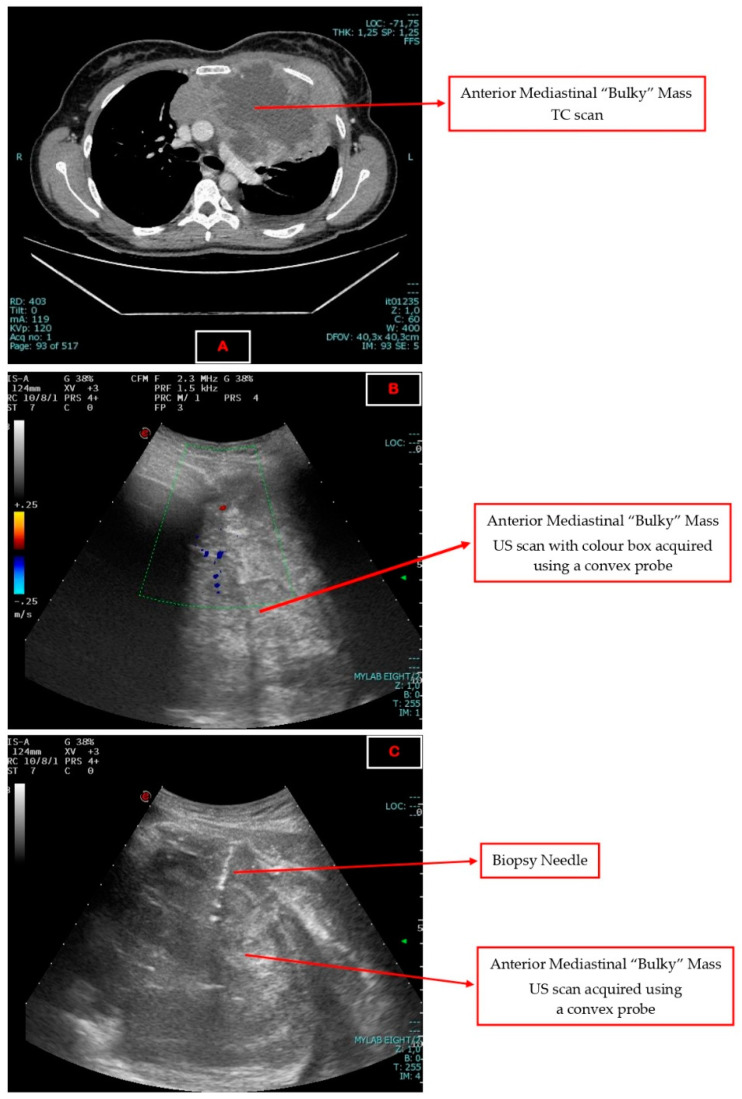
Explanatory image of the procedures. (**A**) An anterior mediastinal mass visualized using TC scan. (**B**) The same lesion visualized using a US-convex probe. (**C**) The biopsy procedure of the same mass performed using US–TCNB technique.

Technical information concerning the biopsy procedure—needle size, number and measurement of collected samples—were also recorded. A US system (Philips iU22, Esaote MyLabEight) equipped with convex, micro-convex and linear probe, characterized by B-Mode 2D imaging modes and associated with Colour and Pulsed Doppler, was used. A convex probe at 3.5–7.5 MHz was mainly used, while the needle calibre varied between 12 and 18-gauge (G) depending on mass and patient characteristics. The most common acoustic windows were located at the intercostal, parasternal and supraclavicular level. An explanatory image of the procedures is shown in Figure 1.

B-mode ultrasound and Doppler modules allowed the accurate characterization of mass location, size, depth, US pattern, vascularization and anatomical relationship with close structures. Mediastinal lesions were classified according to ITMIG classification, proposed in 2017 by the International Thymic Malignancy Interest Group, a tri-compartmental system of subdivision of the mediastinum using transverse CT scans [25].

Informed consent was signed by all patients before the US–TCNB. All biopsies were performed by a thoracic surgeon and an internist with wide US-expertise. All procedures were carried out on an outpatient basis.

During the procedure, patients were placed in a supine position with the neck hyperextended. After skin disinfection, a peripheral venous access was placed and multiparametric monitoring was applied. Operators were equipped with a surgical mask and cap, sterile gowns and gloves. The entire procedure was carried out using disposable sterile devices for both the operating field and instrumentation, including sterile surgical drapes, probe covers and ultrasound gel. For all patients, local anaesthesia was administered: 10–20 mL of 2% lidocaine buffered with sodium bicarbonate at a 9:1 ratio.

Biopsies were performed by freehand US-guided technique and “in plane” mode (needle parallel to scanning axis). This method gave the two operators—one maneuvering the ultrasound probe and the other taking the biopsy sample—continuous visualization of the needle direction for the duration of the procedure. This technique allowed the needle trajectory to be adjusted in “real time” to avoid excessive extra-lesion penetration or the accidental puncture of the lung, vessels and other mediastinal structures.

The automatic collection of tissue samples was performed using Tru-Cut technology and needles ranging from 12 to 18 G. Usually, needles were driven into the mass periphery to avoid the central areas, which often encase necrotic tissue. The number of biopsy frustules depended on the quality of the tissue. The collected frustules were then wrapped in sterile gauze, soaked in normal saline solution, placed in a sterile container for biological samples and carefully labelled before being sent to the hospital anatomo-pathology service for histopathological examination, which was carried out using haematoxylin-eosin staining associated with immunohistochemical evaluation for the main tumour markers at the discretion of the anatomo-pathologist. This survey made possible a definitive diagnosis.

After the procedure, patients were observed for 4 h to identify the early onset of any complications. The diagnostic accuracy, sensitivity and specificity of US–TCNB were estimated by standard formulas as follows:

Accuracy = diagnostic procedures/performed procedures
Sensitivity = true positives/(true positives + false negatives)
Specificity = true negatives/(false negatives + true negatives)

Statistical analysis was performed using commercially available software (Microsoft Excel 365, Microsoft Corp.). Data were reported as absolute numbers/percentages or means ± standard deviation. All data were processed using methods of descriptive statistics.

The study was conducted in accordance with both the Helsinki Declaration and Good Clinical Practice Guidelines although it did not require approval by the Ethics Committee of Città della Salute e della Scienza di Torino. In fact, this retrospective observational study involved patients who attended Molinette Hospital through the National Health System and who had undergone the required procedures with devices and techniques used in normal clinical practice. To ensure personal awareness and privacy, all patients signed written informed consent and their data were processed anonymously.

## 3. Results

**Figure 2:**
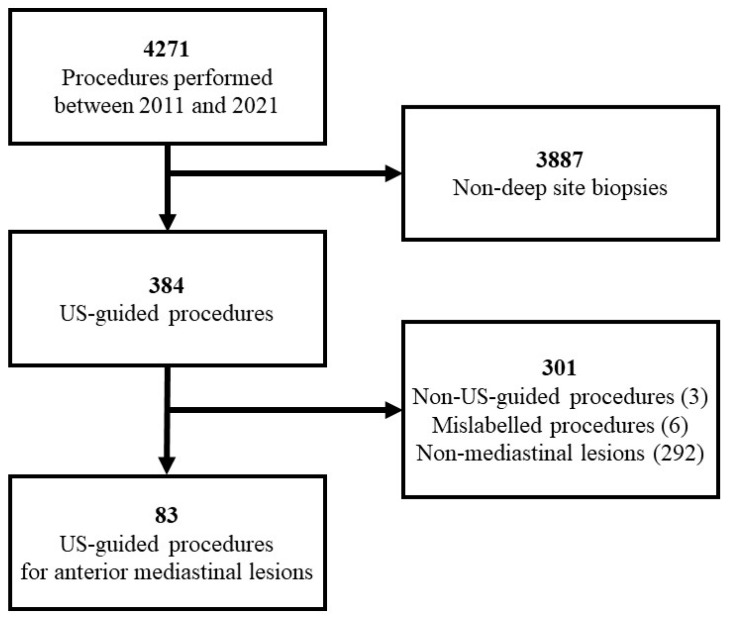
Decision tree showing the selection process of procedures included in the study.

Of the 4271 patients attending the Interdepartmental Unit of Internal and Interventional Ultrasound between 2011 and 2021, 83 were included in the study. The decision tree of patient selection is shown in Figure 2.

Of these patients, 41 (49.4%) were male and 42 female (50.6%), with a mean age of 43.1 ± 19.1 years. As the sample age was not homogeneous, patients were grouped into two age classes: <40 years (*n* = 40) and ≥40 years (*n* = 43). This stratification, in conjunction with sex, afforded a better comparison of results with the literature since these are the two main elements determining the epidemiology of mediastinal pathologies. Patient stratification on the basis of age and sex is shown in Table 1.

**Figure 3:**
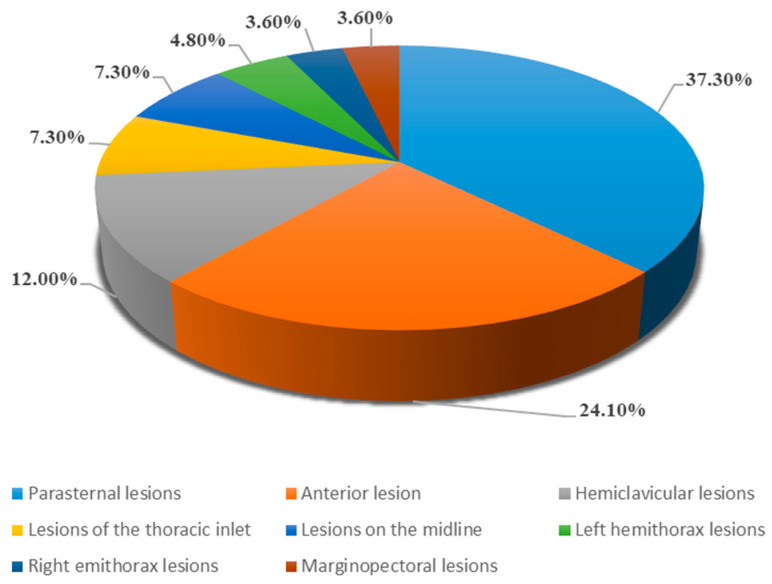
Localization of mediastinal lesions according to ITMIG Classification.

Mediastinal lesions were classified according to the International Thymic Malignancy Interest Group (ITMIG) classification. The most frequent localization was at the right or left parasternal level (*n* = 31), followed by masses in the anteriormost portion of the mediastinal compartment (*n* = 20) and along the hemiclavicular line (*n* = 10, 12.0%). Six patients showed lesions localized at the level of the thoracic inlet and an equal number at the midline level. The exact localization of all investigated masses is shown in Figure 3.

Needles with Tru-Cut technology and automatic sample collection were used for the procedure. Needle size was chosen on the basis of the patient’s age and physical structure and mass characteristics: dimension, position, vascularization. The caliber varied from 12 to 18 gauge: 12G (*n* = 22), 14G (*n* = 10), 16G (*n* = 47) and 18G (*n* = 3). On average, the number of frustules obtained from each procedure was 3.4 ± 1.5. In 7 patients, only 1 frustule was collected, while in other cases, 2 or more samples were taken. Table 2 shows the relationship between needle size and number of collected frustules.

Histological examination was performed in 78 cases with an overall diagnostic accuracy of 94.0% (78/83). US–TCNB showed a sensitivity of 94% and a specificity of 100%. Final diagnoses stratified according to age and sex are reported in Table 3. These included: non-Hodgkin’s lymphoma (32), Hodgkin’s lymphoma (*n* = 21), thymoma (*n* = 9), neuroendocrine tumors (*n* = 4), germ cell tumors (*n* = 3), thyroid cancer (*n* = 1) and other neoplasms (*n* = 8).

In 5 patients (6.0%), the procedure did not lead to a diagnosis. For these, an 18G needle was used in 2 cases (2.4%), a 16G in 1 (1.2%) and a 14G in 2 (2.4%). Afterwards, the US–guided procedure was repeated in three patients, two of which were diagnosed with extensive necrotic cancer.

Complications occurred in 2 patients (2.4%). One experienced mild haemoptysis, but a chest CT angiography and bronchoscopy did not show acute bleeding. The other patient presented symptomatic sinus bradycardia at 40 bpm, without US evidence of pneumothorax, pleuro-pericardial effusion or alterations of the left ventricular systolic function. In this case, the symptoms resolved rapidly after intravenous administration of 0.5 mg atropine, indicating bradycardia induced by vagal hyper tone.

US–TCNB procedure costs were estimated at EUR 120.00 and included all devices used to perform the biopsy: sterile gowns and gloves, disinfection materials, sterile field, local anaesthesia, syringes, probe covers, and cutting needle:. The costs for ultrasound equipment and staff remuneration were not considered.

## 4. Discussion

Anterior mediastinal lesions are quite rare and are often categorized as malignant tumours [1]. Thus, a prompt diagnosis is essential for a favourable patient prognosis.

As mentioned above, clinical presentation and laboratory tests are not always sufficient to confirm a diagnosis, making it necessary to perform a histological examination. At present, international guidelines, such as those of the European Society for Medical Oncology (ESMO) and Italian Association of Medical Oncology (IAOM), indicate surgical biopsy as the “gold standard” method [11,12,13,14].

However, in recent years, clinical interest has focused on other percutaneous techniques like Ultrasound-guided Transthoracic Core Needle Biopsy. Therefore, the aim of the study was to evaluate the effectiveness of this method with regard to lesions of the anterior mediastinum.

A total of 83 patients undergoing US-TCNB were included in the analysis. In 78 of them, histological examination on the material obtained by the US-guided biopsy was able to define the diagnosis of the lesion correctly, with an overall accuracy of 94.0%. These results are in line with those of previous studies. In particular, diagnostic accuracy in the present study, despite the smaller sample size, was similar to that reported by Huang et al., Petkov et al., and Navin et al.: accuracy, 94% vs. 95–96%; sensitivity, 94% vs. 94–95%; specificity, 100% vs. 100%) [18,19,24]. It was also better than the results reported by Chen et al., who investigated a similar size population: accuracy, 94% vs. 89%; sensitivity, 94% vs. 71%; specificity, 100% vs. 84%) [24]. Outcomes comparison between the present study and previous works are shown in Table 4.

US–TCNB appeared to maintain a competitive diagnostic accuracy compared to other biopsy techniques, both surgical and percutaneous. Its diagnostic values were comparable to those of more invasive surgical procedures—Anterior Mediastinotomy and Video-Assisted Thoracoscopy (94 vs. 96 and 98%, respectively) [16,26]—and percutaneous techniques—Trans-Bronchial Needle Aspiration, CT-guided core needle biopsy and MRI-Guided Biopsy (94 vs. 93, 94 and 88%, respectively) [22,27,28].

In the present investigation, US–TCNB failed to diagnose only 5 patients. However, when the procedure was repeated, two of those patients were diagnosed with extensive necrotic cancer. It is therefore likely that the necrotic tissue interfered with obtaining a suitable sample during the first biopsy [29]. Curiously, 2 of the undiagnosed samples were taken with a 18G needle, which was used in only three patients. Thus, the diagnostic accuracy of the 18G needle biopsy would appear limited in our sample although its small size did not allow for a definitive conclusion. Moreover, previous studies performed on wider populations did not report a significative influence from the needle gauge in attaining nondiagnostic biopsies [18,24]. Certainly, further studies in this regard are needed to clarify the possible role of needle size on US–TCNB accuracy.

The histological analyses assisted in classifying the mediastinal lesions. As proposed by Carter et al. [3], the sample population was stratified on the basis of sex and age. Interestingly, the frequency and distribution of the main pathologies of the anterior mediastinum gave similar results to those reported in the literature [3].

Regardless of sex, all thymomas (*n* = 9), neuroendocrine carcinomas (*n* = 4) and neoplasms classified as “other”, were diagnosed in patients over 40 years. The latter included plasmocytomas (*n* = 2), mesotheliomas (*n* = 2), lung cancer (*n* = 1), primary neuroectodermal tumour (pNET) (*n* = 1), T-lymphoblastic leukaemia (*n* = 1) and necrotic carcinoma (*n* = 1). On the other hand, there were differences between the sexes among youngsters: germ cell cancer was prevalent in males, while haematological neoplasia such as Hodgkin’s and non-Hodgkin’s lymphomas were more frequent in females.

The periprocedural complication rate for US–TCNB in this study was 2.4%. Of these 2 patients, one experienced mild haemoptysis while the other was affected by sinus bradycardia, both of which can be classified as minor complications. This result was in line with other reports in the literature. In fact, Huang et al., and Navin et al., observed similar minor complications rates (4 and 2%, respectively) [19,24], while Petkov et al., did not differentiate complication severity and only reported an overall incidence of 2.4% [18]. As the complications in both our patients were not severe, they did not lead to hospitalization. This fact is notable because it allowed the biopsy to be performed in an outpatient setting. The need for hospital admission is otherwise always necessary for surgical techniques, such as Anterior Mediastinotomy and VATS. Hospitalization requirement leads not only to a rise in procedure cost, but also to an increased risk of complications including nosocomial infections. In fact, compared to other surgical techniques, US–TCNB caused fewer minor complications (2.4 vs. 10 and 33% for VATS and Mediastinotomy, respectively) [16,26]. Major complications were not observed in our cohort, which was also in line with those of previous investigations. In fact, major complications were only reported by Navin et al., who experienced a symptomatic pneumothorax requiring intervention [24]. On the other hand, major complications are more frequent in cases of surgical procedures, where they may even require a transfer to the intensive care unit especially in the presence of comorbidities, more fragile patients, or in those with a voluminous mediastinal mass that compresses the respiratory tract [18].

Economic aspects are certainly not of secondary importance. Although in the present study we could only make a rough estimate of US–TCNB costs, our calculations confirmed the data in the literature. Needle biopsy costs are on average 10–15 times lower than VATS and other surgical techniques [30]. This fact is mainly due to the outpatient setting and to lower complication rates, which account for 13% of total costs [21].

Although this study was one of the few on the matter, it had some limitations that are worth noting. First, the retrospective nature of the study, relatively small sample size (also due to the monocentric setting) and lack of a control group may introduced selected bias or difficulty in data analysis. Moreover, the limits of the US–TCNB technique should also be considered, such as the operator dependency or the possible absence of optimal acoustic windows for visualizing the lesion.

If the findings this study are confirmed, they could affect both healthcare policy and clinical guidelines. In fact, a US-guided needle biopsy could be proposed for all patients having a suitable anterior mediastinal mass before the surgeon is called. Such a recommendation would benefit both the patient and healthcare system: the former would undergo a less invasive technique, while the latter could save money because of lower operating costs.

## 5. Conclusions

Based on the presented data, US-guided needle biopsy can be considered as an extremely effective and safe method for the histological diagnosis of anterior mediastinal pathologies. Its effectiveness resulted comparable to the main surgical procedures, but it was characterized by a lower complication rate and lower costs.

Of course, further studies are needed not only to confirm these outcomes, but also to investigate the effectiveness of US–TCNB in different patient populations and compare it with other percutaneous diagnostic procedures such as trans-bronchial needle aspirations, CT-guided core needle biopsy, and MRI-guided biopsy.

## Figures and Tables

**Table 1:** Patient stratification on the basis of sex and age.

	<40 Years Old (*n*, %)	≥40 Years Old (*n*, %)	Total (*n*, %)
Males (*n*, %)	20 (24.1)	21 (25.3)	41 (49.4)
Females (*n*, %)	20 (24.1)	22 (26.5)	42 (50.6)
Total (*n*, %)	40 (48.2)	43 (51.8)	83 (100.0)

**Table 2:** Relationship between needle size and number of collected frustules.

Needle Size	Collected Frustules (*n*, %)						
	1	2	3	4	5	>5	Total
12G	3 (13.6)	7 (31.8)	10 (45.5)	1 (4.5)	0 (0.0)	1 (4.5)	22 (100.0)
14G	1 (10.0)	1 (10.0)	4 (40.0)	3 (30.0)	1 (10.0)	0 (0.0)	10 (100.0)
16G	3 (6.4)	3 (6.4)	16 (34.0)	15 (31.9)	6 (12.8)	4 (8.5)	47 (100.0)
18G	0 (0.0)	0 (0.0)	0 (0.0)	2 (66.6)	1 (33.3)	0 (0.0)	3 (100.0)
Total	7 (8.4)	11 (14.5)	29 (34.9)	21 (25.3)	8 (9.6)	5 (6.0)	82 (100.0) *

* Needle size information was not available for one patient.

**Table 3:** Final diagnoses stratified according age and sex.

Final Diagnosis	Males		Females		Total
	<40 y (*n*, %)	≥40 y (*n*, %)	<40 y (*n*, %)	≥40 y (*n*, %)	
Non-Hodgkin’s lymphoma	13 (15.7)	3 (3.6)	8 (9.6)	8 (9.6)	32 (38.6)
Hodgkin’s lymphoma	4 (4.8)	2 (2.4)	10 (12.0)	5 (6.0)	21 (25.3)
Thymoma	0 (0.0)	5 (6.0)	0 (0.0)	4 (4.8)	9 (10.8)
Neuroendocrine tumors	0 (0.0)	4 (4.8)	0 (0.0)	0 (0.0)	4 (4.8)
Germ cell tumors	2 (2.4)	1 (1.2)	0 (0.0)	0 (0.0)	3 (3.6)
Thyroid cancer	0 (0.0)	1 (1.2)	0 (0.0)	0 (0.0)	1 (1.2)
Other	0 (0.0)	5 (6.0)	0 (0.0)	3 (3.6)	8 (9.6)
Undiagnosed	1 (1.2)	0 (0.0)	2 (2.4)	2 (2.4)	5 (6.0)
Total	20 (24.1)	21 (25.3)	20 (24.1)	22 (26.5)	83 (100.0)

**Table 4:** Comparation between available study on US-TCNB for mediastinal lesions diagnosis.

Reference	Country	Study Design	Sample Size (*n*)	Accuracy (%)	Sensitivity (%)	Specificity (%)	Complications (%)
Chen et al., 2014 [22]	China	Retrospective	80	89	71	84	Major complications: 0
							Minor complications: NR
Huang et al., 2019 [19]	China	Retrospective	121	95	94	100	Major complications: 0
			(10 AMMs)				Minor complications: 4
Petkov et al., 2020 [18]	Bulgaria	Retrospective	308	96	95	100	Overall complications: 2.6
			(283 AMMs)				
Navin et al., 2021 [23]	USA	Retrospective	337	96	-	-	Major complications: 0.3
			(303 AMMs)				Minor complications: 2
Vischia et al., 2023	Italy	Retrospective	83	94	94	100	Major complications: 0
							Minor complications: 2.4

AMMs: Anterior Mediastinal Masses; NR: Not Recorded.

## Data Availability

The data presented in this study are available on request from the corresponding author. The data are not publicly available due to privacy restrictions.
